# A dataset of skin lesion images collected in Argentina for the evaluation of AI tools in this population

**DOI:** 10.1038/s41597-023-02630-0

**Published:** 2023-10-18

**Authors:** María Agustina Ricci Lara, María Victoria Rodríguez Kowalczuk, Maite Lisa Eliceche, María Guillermina Ferraresso, Daniel Roberto Luna, Sonia Elizabeth Benitez, Luis Daniel Mazzuoccolo

**Affiliations:** 1 Departamento de Informática en Salud, Hospital Italiano de Buenos Aires, Tte. Gral. Juan Domingo Perón 4190, 1199, Ciudad Autónoma de, Buenos Aires, Argentina; 2 Universidad Tecnológica Nacional, Av. Medrano 951, 1179, Ciudad Autónoma de, Buenos Aires, Argentina; 3 Servicio de Dermatología, Hospital Italiano de Buenos Aires, Tte. Gral. Juan Domingo Perón 4190, 1199, Ciudad Autónoma de, Buenos Aires, Argentina; 4 Instituto de Medicina Traslacional e Ingeniería Biomédica (IMTIB), UE de triple dependencia CONICET- Instituto Universitario del Hospital Italiano (IUHI) - Hospital ITaliano (HIBA), Tte. Gral. Juan Domingo Perón 4190, 1199, Ciudad Autónoma de, Buenos Aires, Argentina; 5 Instituto Universitario del Hospital Italiano, Potosí 4265, 1199, Ciudad Autónoma de, Buenos Aires, Argentina

**Keywords:** Medical imaging, Skin cancer

## Abstract

In recent years, numerous dermatological image databases have been published to make possible the development and validation of artificial intelligence-based technologies to support healthcare professionals in the diagnosis of skin diseases. However, the generation of these datasets confined to certain countries as well as the lack of demographic information accompanying the images, prevents having a real knowledge of in which populations these models could be used. Consequently, this hinders the translation of the models to the clinical setting. This has led the scientific community to encourage the detailed and transparent reporting of the databases used for artificial intelligence developments, as well as to promote the formation of genuinely international databases that can be representative of the world population. Through this work, we seek to provide details of the processing stages of the first public database of dermoscopy and clinical images created in a hospital in Argentina. The dataset comprises 1,616 images corresponding to 1,246 unique lesions collected from 623 patients.

## Background & Summary

Recent years have witnessed an increase in the development of Artificial Intelligence (AI) algorithms for automated medical image analysis. One of the medical specialties in which AI has been widely explored is dermatology^1^, where specialists heavily rely on visual analysis, and different imaging modalities (dermoscopy and clinical imaging) are commonly used during clinical examination^2–4^. In this context, researchers have demonstrated that a convolutional neural network, an algorithm commonly used for automatic image processing, could achieve performance on par with specialists in skin cancer identification^5^. Moreover, a systematic review and meta-analysis of studies comparing the outcomes of AI models and those of healthcare professionals, concluded that the diagnostic performance of AI models was equivalent to that of caregivers in several specific use cases^6^.

The development and assessment of AI algorithms have been made possible in large part by the work of the scientific community to build and make public high-quality databases^7–9^. However, several studies have pointed out the need for more transparency and completeness in reporting demographic information in these databases^10–12^ and in describing AI developments^13^. Furthermore, a systematic review of the major dermatologic imaging databases used for AI research^14^ revealed an uneven geographic distribution and thus limited representation of the diversity of the global population, which may result in what is referred to as health data poverty. This concept describes the hindrance that specific individuals or groups face in benefiting from innovations and research due to the scarcity of representative data^15^.

For multiple reasons, this situation has been pointed out as problematic by the medical image computing (MIC) community. First, it is known that training algorithms with databases made up of a few specific groups defined by attributes such as sex, age, origin, ethnicity or socioeconomic level, has given rise to what is known as algorithmic bias, also characterized by low performance of these models in the case of minorities^16^. At a time when there is enormous interest in implementing clinical decision support systems (CDSS) to assist clinicians, the need to ensure algorithmic fairness has risen to the top of the agenda since the introduction of tools that work inadequately for some people may constitute a potential harm^17^.

On the other hand, there is evidence that the performance of AI algorithms decreases when tested on populations different from those used to train them^18^. In this sense, it is not possible to evaluate the performance of the methods or for them to learn specific characteristics of those sub-populations that are not represented in the databases. As a consequence, when the developments are intended to be deployed in left-out populations, the creation of new datasets, usually isolated and unpublished, becomes necessary^19^.

Based on the above, our team sought to collaborate with two lines of action. First, designing a skin lesion image database that includes metadata using the criteria defined by the International Skin Imaging Collaboration (ISIC), which aims to promote and facilitate the use of digital skin imaging to combat skin cancer. We accompany the database with this article, which aims to provide detailed information on the processes of design, compilation, and refinement of this database, as well as the description of its final content, to ensure the necessary transparency demanded by the community through different initiatives, guidelines, and frameworks^20–23^. Secondly, our greatest contribution lies in publishing the first anonymized dataset of clinical and dermoscopy images of skin lesions collected entirely in Argentina and the first of its kind in Hispanic America. The database was meticulously audited by experts from the Department of Dermatology of Hospital Italiano de Buenos Aires (HIBA), a highly complex hospital located in Argentina, and following the recommendations found in the literature. A similar project was conducted by the neighboring country of Brazil, in which they published a carefully refined database of clinical images^24^. However, the sociodemographic and epidemiological differences between our populations encouraged the conduct of this work to ensure the representativeness of the Argentinean people and their particularities in dermatological image archives. We also hope to encourage other Latin American countries to become involved in this type of endeavor to facilitate the translation of new technologies to clinical settings in the region.

The dataset described here was composed of information collected from 623 patients seen by expert dermatologists at HIBA. We included 1,616 images (1,270 contact-polarized dermoscopy images and 346 clinical images) captured from 1,246 lesions corresponding to the most frequent diagnoses observed at the institution.

## Methods

The Institutional Review Board of HIBA approved the studies to develop AI models for skin lesion classification and evaluate them using a local database (Approval No. 5918 and Approval No. 5930), following which the release of the de-identified local dataset was approved. The IRB approved the publication of the images along with the annotations under a Creative Commons Attribution (CC BY) license, which allows reusers to distribute, remix, adapt, and build upon the material in any medium or format, so long as attribution is given to the creator. Because this study used secondary databases and did not modify the usual practice of the patients involved, the IRB waived the right to consent. Data preparation was carried out in compliance with the data circuit to ensure de-identification and the protection of personal data.

HIBA is a community-based tertiary care hospital in Buenos Aires, Argentina. This project was conducted by the Dermatology Department and the Artificial Intelligence and Data Science in Health program of the Health Informatics Department of the institution.

The following subsections describe the steps involved in the construction of the database.

### Data collection

To populate the database we retrieved from the institutional records information collected between 2019–2022 regarding patients with common skin lesions who consulted the Dermatology Department, as well as clinical and dermoscopy images acquired at the time of the consultation. The cases included in the database were selected by three expert dermatologists.

During the anamnesis, the specialists asked patients about their personal and family history of melanoma and other personal data. In addition, they recorded the patient’s skin tone employing the Fitzpatrick scale^25^, based on the subject’s physical characteristics. A visual examination of the patient’s skin lesions was performed, and photographs of clinically relevant lesions were acquired. Clinical images were taken through the smartphones of the respective professionals. In contrast, dermoscopy images were acquired using a video microscope VL-7EX II (Scalar Corporation, Tokyo, Japan) and Dermagraphix Mirror 7 (Canfield Scientific, New Jersey, United States of America), a Camera Medicam 1000 s and Vexia with FotoFinder Universe 2 (FotoFinder Systems, Bad Birnbach, Germany), a camera Medicam 800Hd attached to a dermoscope manual tower with FotoFinder 2007 (FotoFinder Systems, Bad Birnbach, Germany) or by resorting to a dermoscopic attachment for smartphones.

The anatomical location and presumptive diagnosis were recorded for each lesion. In case of suspicion of malignancy, the professional indicated the removal of the lesion by surgical procedure and its subsequent histopathological analysis. The definitive diagnosis of these lesions was defined after knowing the result delivered by the Department of Pathology of the institution.

### Data selection

The patients’ personal and clinical information, as well as the biopsy results, were validated with the data recorded in the EHR. This information and the images were uploaded to a REDCap platform version 10.6.7 (https://www.project-redcap.org/) to form the database. In this regard, patients with at least one photographed skin lesion corresponding to malignant melanoma (MM), basal cell carcinoma (BCC), squamous cell carcinoma (SCC), actinic keratosis (AK), melanocytic nevus (NV), seborrheic keratosis (SK), solar lentigo (SL), lichen planus-like keratosis (LK), dermatofibroma (DF) or vascular lesions (VASC) were included. Cases of suspected malignant lesions without histopathological analysis performed at the institution were excluded.

### Data processing

Data processing was performed by experienced dermatologists and biomedical engineers at the institution. Recommendations outlined in HIPAA^26^ were followed to recognize those identifiers of individuals necessary to remove to achieve a “safe harbor” method of de-identification. To this end, all images underwent an additional review to discard or crop those photographs of the face and complete anatomical sections including tattoos or other potential identifiers. In the presence of multiple images of the same lesion acquired during the same episode, the reviewers chose one or more photographs taking diagnostic representativeness and image quality into account. Finally, those images where it was impossible to identify the lesion due to low resolution, artifacts, out-of-focus lesions, or lesions occluded by hair or markings, were deleted.

Regarding the metadata (personal and clinical information of the patient and additional information on the lesion), each of the records in the REDCap platform was reviewed and contrasted against the institutional records to identify inconsistencies. In each case, age was approximated to the date of image acquisition and rounded to 5-year intervals to ensure data de-identification. The fields were identified as missing data in case of missing, incorrect, or doubtful information.

Each patient and image was assigned a consecutive and unique alphanumeric identifier. The lesions were numbered sequentially for each patient. Finally, all information was translated from Spanish to English.

## Data Records

### Repository and dataset format

The dataset described here is permanently accessible to the public through the ISIC Archive^27^ at the following link 10.34970/587329 and was released under a Creative Commons Attribution (CC BY) license. The images, as well as a metadata table, can be downloaded from the repository. However, since it is not possible to access the unstructured data in this way, we have included this same data table with additional information regarding skin type as a Supplementary Table.

Images were uploaded to the archive in JPEG format and the associated metadata was shared via a comma-separated values (CSV) file. Each record in the metadata file contained a file name, a unique lesion identifier, and an anonymized patient identification number. Regarding the patient, personal and family history of melanoma, biological sex, approximate age at the time of image acquisition, and skin tone were also included. In addition, the corresponding diagnosis, the method by which it was confirmed, a benign/malignant label, and the general anatomical site where the lesion was found were incorporated. Finally, it was indicated whether it was a clinical or dermoscopy image, the type of dermoscopy if applicable, and the geographic region in which this dataset was formed.

### Dataset description

The original set of 1,755 records was thoroughly reviewed and 139 images were eliminated in this process. As a result, 43 lesions and 26 patients were excluded from the collection. The final database consisted of 1,616 images, of which 1,270 cases were contact-polarized dermoscopy images and 346 were clinical images. Each patient could have more than one skin lesion and several images per skin lesion. The dataset was formed with data collected from 623 unique patients and 1,246 unique lesions.

All patients included in the database had to have attended at least one medical consultation at the hospital located in Buenos Aires, Argentina. The age was not recorded for a percentage of less than 1% of the total patients, and the mean age was 62.1 ± 17.3 years old. Regarding sex, this information was not recorded for only two patients, while the rest of the cohort corresponded to 339 females and 282 males.

It was considered important to incorporate skin tone information, as this data could be retrieved from the institutional records for 566 patients (I: 44, II: 451, III: 66, IV: 5). This implied that the skin tone is detailed for more than 93% of the records (images) that make up the database.

In relation to the personal and/or family history of MM, multiple missing records were found, reaching 333 patients for whom neither of these two fields was recorded. With regard to personal history of MM, it was found that in 54.41% of the cases this data was not reported, while in 24.08% there were previous findings of MM and in the remaining 21.51% there were none. On the other hand, for 61.96% of the patients there was no information about a family history of MM, although of the remaining proportion, 4.98% acknowledged having family members with identified MM and 33.06% denied the existence of a family history of this type. Only 1.12% of the dataset cases had a positive personal and family history of MM.

**Fig. 1:**
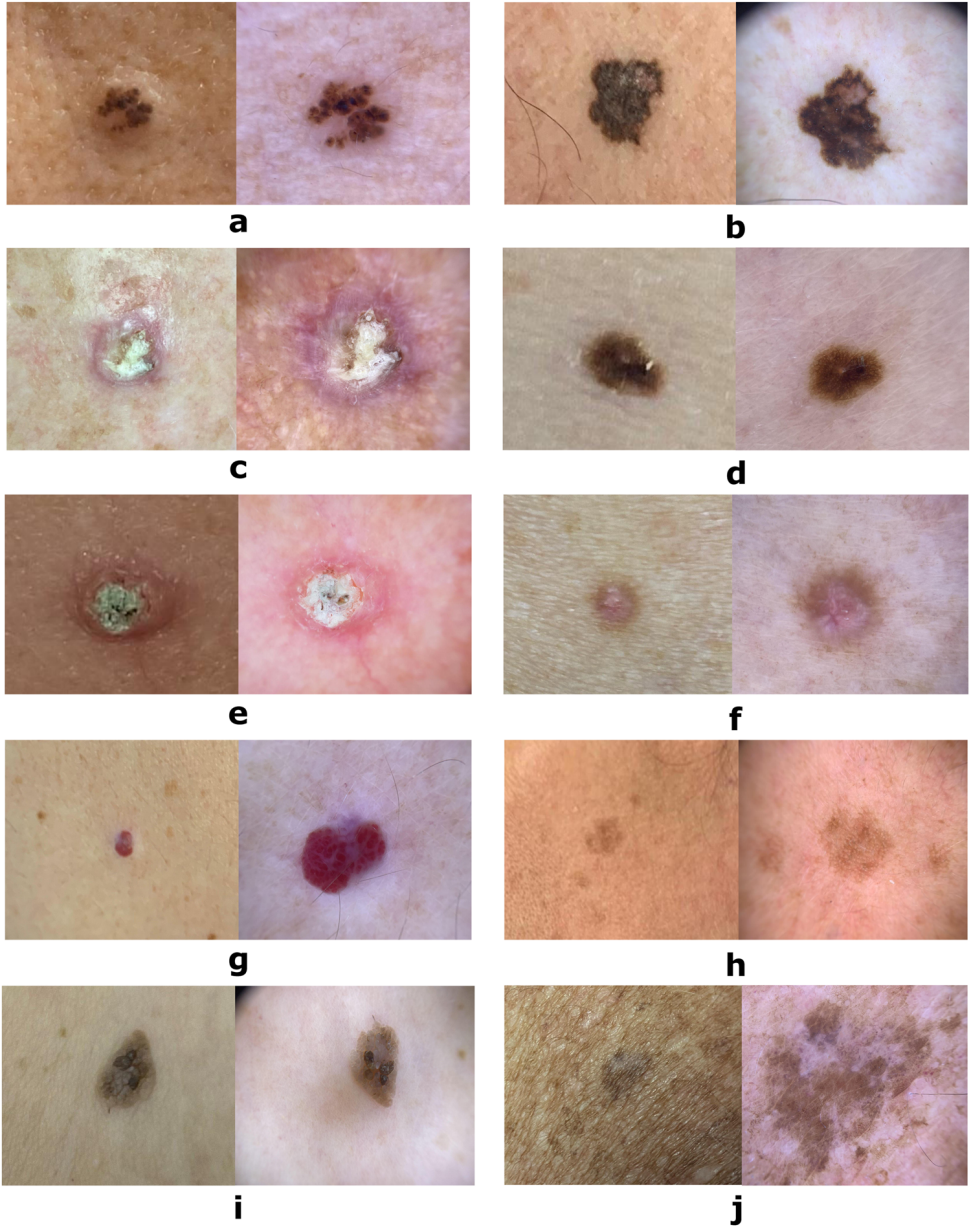
Skin lesion images samples from the dataset. (**a**) Basal Cell Carcinoma, (**b**) Melanoma, (**c**) Squamous Cell Carcinoma, (**d**) Melanocytic Nevus, (**e**) Actinic Keratosis, (**f**) Dermatofibroma, (**g**) Vascular Lesion, (**h**) Solar Lentigo, (**i**) Seborrheic Keratosis, (**j**) Lichen planus-like Keratosis. For each diagnosis, a clinical image (left) and a dermoscopy (right) of the same lesion are presented.

Each patient was represented by at least one lesion, with the mean number of lesions per patient being two, and with one patient reaching a maximum of 63 lesions included in a study. Likewise, each lesion had at least one dermoscopy or clinical image, with cases in which both images were available. The maximum number of dermoscopies captured for a lesion was five, while the upper limit for clinical photographs was two. In general, dermoscopies were recorded more often than clinical images, as a consequence of the usefulness of the formers for the diagnosis of skin lesions^28^. The number of lesions, image type distribution and percentage of biopsied lesions concerning the different diagnosis are depicted in Table 1. Samples of each type of image for these diagnoses are shown in Fig. 1.

**Table 1:** Number of lesions and images for each type of skin lesion.

Skin lesion	# lesions	% biopsied lesions	# dermoscopy images	# clinical images	#total images
Basal Cell Carcinoma	235	100%	228	112	340
Squamous Cell Carcinoma	91	100%	111	47	158
Malignant Melanoma	172	100%	194	59	253
Melanocytic Nevus	556	27.69%	549	53	602
Actinic Keratosis	44	54.55%	46	17	63
Dermatofibroma	41	31.71%	39	22	61
Vascular Lesion	43	20.93%	41	10	51
Solar Lentigo	15	40%	14	4	18
Seborrheic Keratosis	48	43.75%	47	21	68
Lichenoid Keratosis	1	0%	1	1	2
**total**	**1,246**	**58.18%**	**1,270**	**346**	**1,616**

Regarding the location of the lesion, eight general anatomical sites were reported: lateral, anterior and posterior torso, lower and upper extremities, head/neck, palms/soles and oral/genital. Melanocytic lesions (MM and NV) were found to be mainly concentrated in the posterior torso, anterior torso and lower extremities, while BCC, SCC, AK and SL were most frequently located in the head or neck. Almost all of the DF were from the lower extremities, while the majority of VASC and SK came from the anterior torso in addition to the head/neck region. The set contains one LK lesion, which was photographed on the posterior torso.

## Technical Validation

All images and lesions included in the dataset were thoroughly reviewed by dermoscopy experts. The histopathological analysis results report determined the definitive diagnosis of all malignant lesions. Moreover, for benign lesions there was a variable percentage of diagnoses confirmed in this way. Non-biopsied lesions were labeled based on the evaluation of the treating dermatologic expert or consensus among a group of professionals. Ultimately,

58.18% (N = 725) of skin lesions in the dataset were biopsy-proven, which corresponds to 1,036 images or 64.11% of the complete collection. This resembles the values found in other datasets such as PAD-UFES-20^24^ and HAM10000^8^, with 58.4% (N = 1,342) and 53.13% (N = 6,227) images with biopsy confirmation, respectively. Melanoma *in situ* and invasive melanoma were both identified as melanoma. Although actinic keratosis is often considered a precursor of squamous cell carcinoma, in the dataset this lesion was labeled as benign.

Practitioners uploaded the metadata during patient anamnesis and subsequently retrieved it from the information recorded in the EHR. Of these data, those with the possibility of varying over time (age and history) were approximated to the date of acquisition of the image included in the dataset. On the other hand, in the vast majority of cases, skin tone was recorded by the dermatology specialist in charge of the patient considering skin tone, eye tone and the patient’s own responses to the skin’s reaction to sun exposure. In a low percentage of cases where this information was not recorded in the institutional records, dermoscopy experts evaluated the sufficiency of the dermoscopy image to infer this information from the characteristics and colors of the melanocytic nevi^29,30^.

## Usage Notes

To our knowledge, this is the first database of clinical and dermoscopy images of skin lesions collected and made publicly available by a highly complex hospital in Hispanic America. While the database can be combined with larger image collections and used to train AI algorithms, its use is valuable in evaluation and validation processes, as well as for the comparison of different AI systems.

This initiative arose from the interest in implementing AI tools as support systems in institutions in our country and from the need to validate in our population algorithms created with databases from countries in North America, Europe or Oceania^8,9,14,31–33^. The underrepresentation of South American, African and Asian populations in international databases may be the result of the lack of funding for research, the time required to collect and process these data, the demand in terms of human resources, ethical and regulatory aspects, as well as technological limitations in some health centers^14,16,17^. There are more complex systemic and structural reasons, such as restricted access to the health system and deep economic inequalities^16,17^, which call for different agencies to take measures to avoid falling into health data poverty. This situation is more pronounced in countries classified as lower-middle and lower-income economies.

Through this project, we intend to make this situation visible, promote the construction and open access publication of databases from different regions of the world, and encourage involvement with international efforts such as the ISIC Archive. We also hope to contribute to the MIC community investigating problems such as domain shift and algorithmic bias.

The dataset shares features common to other dermatologic image sets such as the different diagnostic categories collected and their relative frequency, the percentage of lesions with biopsy-proven diagnosis, and the publication of the images without the application of preprocessing except for cropping to facilitate identification of the lesion of interest^8,14,24,32,33^. We consider that the variability in terms of acquisition equipment used, illumination conditions, resolution and the existence or not of artifacts approximates the application context in which these types of tools would be deployed. However, the use of techniques for color normalization and consistency, detection and precise localization of lesions, elimination of artifacts and treatment of class imbalance, among others, is recommended.

Within the limitations, we understand that a selection bias could arise due to the preference to include in this database cases from institutional records that were useful for the purposes of a research and innovation project whose main objective was the development of AI models for use in a hospital located in Buenos Aires, Argentina. In this sense, the images were selected based on the diagnoses of interest, excluding multiple categories usually photographed in the clinical setting and excluding data that could have identifying components such as faces and tattoos. Moreover, as all images are from patients who attended the hospital located in Buenos Aires, this data set does not reflect the diversity of the population throughout the country. This may impact the distribution of the different classes of skin lesions and may not reflect the actual prevalence observed in clinical practice. Furthermore, we are aware that clinical and dermoscopic imaging pairs have not been provided for all lesions incorporated in this set.

Finally, we consider that this is a first step towards the construction of a collaborative database with different medical centers in the country and even with other healthcare providers in the region in order to constitute a truly diverse initiative to try to guarantee our population access to state-of-the-art technology.

### Supplementary information

Supplementary Table

## Data Availability

Python scripts for exploratory data analysis and dataset comparison, as well as supplementary data, are publicly available at https://github.com/piashiba/HIBASkinLesionsDataset.
